# Enhancer RNAs: transcriptional regulators and workmates of NamiRNAs in myogenesis

**DOI:** 10.1186/s11658-021-00248-x

**Published:** 2021-02-10

**Authors:** Emmanuel Odame, Yuan Chen, Shuailong Zheng, Dinghui Dai, Bismark Kyei, Siyuan Zhan, Jiaxue Cao, Jiazhong Guo, Tao Zhong, Linjie Wang, Li Li, Hongping Zhang

**Affiliations:** Farm Animal Genetic Resources Exploration and Innovation Key Laboratory of Sichuan Province, College of Animal Science and Technology, Sichuan Agricultural University, Chengdu, 611130 China

**Keywords:** Enhancer RNA, NamiRNAs, MicroRNA, Myogenesis, Transcriptional regulator

## Abstract

miRNAs are well known to be gene repressors. A newly identified class of miRNAs termed nuclear activating miRNAs (NamiRNAs), transcribed from miRNA loci that exhibit enhancer features, promote gene expression via binding to the promoter and enhancer marker regions of the target genes. Meanwhile, activated enhancers produce endogenous non-coding RNAs (named enhancer RNAs, eRNAs) to activate gene expression. During chromatin looping, transcribed eRNAs interact with NamiRNAs through enhancer-promoter interaction to perform similar functions. Here, we review the functional differences and similarities between eRNAs and NamiRNAs in myogenesis and disease. We also propose models demonstrating their mutual mechanism and function. We conclude that eRNAs are active molecules, transcriptional regulators, and partners of NamiRNAs, rather than mere RNAs produced during enhancer activation.

## Introduction

The identification of lin-4 miRNA in *Caenorhabditis elegans* in 1993 [1] triggered research to discover and understand small microRNAs’ (miRNAs) mechanisms. Recently, some miRNAs are reported to activate target genes during transcription via base pairing to the 3ʹ or 5ʹ untranslated regions (3ʹ or 5ʹ UTRs), the promoter [2], and the enhancer regions [3]. These miRNAs are termed NamiRNAs. In mammals, miRNAs/NamiRNAs control more than half of the protein-coding genes [4]. For example, the 3ʹ UTRs of about 60% of known human protein-coding genes harbor binding sites for miRNAs/NamiRNA [5], serving as docking sites for either activating or inhibiting these genes. Hence, miRNAs (including NamiRNAs) are a significant factor in cellular transcription.

**Fig. 1:**
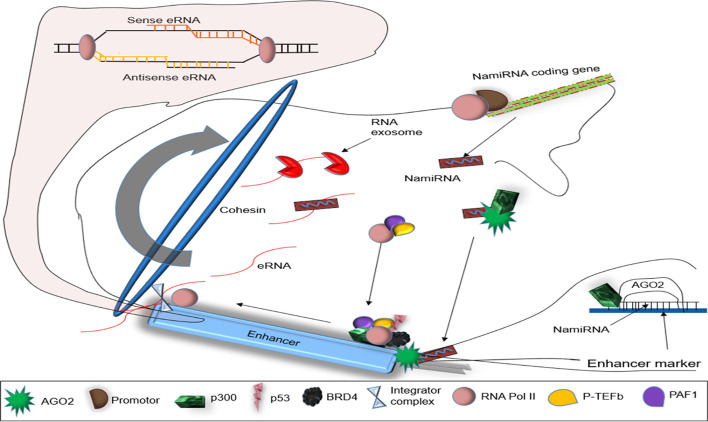
Biogenesis of eRNA. NamiRNA forms a complex with nAGO2 and p300, which activate enhancer markers such as H3K27ac, H3K4me3, and H3K4me1 at active enhancers making the enhancer recognizable to the Pol II. TFs and proteins such as P-TEFb, PAF1, and SPT6 bind to Pol II and other enhancers associated with components like p53 and P300 CBP and BRD4. Pol II is activated following the phosphorylation of Pol II CTD and abundant PAS at the TSS of active enhancers. Pol II bidirectionally transcribes the enhancer and its halter by the integrator complex cleaving the eRNA transcripts

Alternatively, the eRNAs are small non-coding RNA (ncRNA) transcribed by RNA polymerase II (Pol II) from enhancer loci in a similar way as NamiRNA [6]. eRNAs are transcribed as single or double strands (3ʹ to 5ʹ UTR and vice versa) (Fig. 1). But their associated enhancers are not always marked with H3K4me3 (a Pol II epigenetic marker at promoters) [8], which causes a bias transcription. However, enhancers and eRNAs are tissue- and cell-specific [8, 9] and are involved in enhancer mediated transcription and activation [10, 11]. Similar to NamiRNAs, eRNAs have a similar sequence, secondary structures, and some complement regions in their target promoters of the corresponding enhancer [9]. Therefore, they serve as inducing drivers in NamiRNA-enhancer-regulated control [9].

The discovery of eRNAs and NamiRNAs has paved a new path in modern cellular genomics, but the differences and similarities in their mechanisms remain unsolved. Here, we review the regulatory effects of NamiRNAs and eRNAs in cellular transcription and their repercussions in myogenesis, diseases, and therapeutics.

## eRNA and NamiRNA biogenesis

The biogenesis of eRNA and NamiRNAs may co-occur due to their nuclear associated molecular activities in the nucleus. They are transcribed from enhancers and NamiRNAs from miRNA coding genes. However, they end up being close to target enhancers and genes due to chromatin looping and gene activation. NamiRNAs trigger the transcription of eRNAs, and subsequently, the transcribed eRNAs and the NamiRNA activate the target gene and enhancer.

### eRNA originates from an active enhancer

The first enhancer (~ 72 bp) was cloned out of simian virus 40 (SV40), which is associated with an increase in expression of the human β-globin gene [12]. Enhancers and their RNAs are often located in the same loci as their target genes and come to proximity through chromatin looping and restructuring during cellular growth [13] (Fig. 1). They subsequently bind to coactivators and transcription factors (TF) [14] and orchestrate their target gene’s activation [15] (Fig. 1). Most eRNAs are usually unspliced and short, with an average size of 346 nucleotides [16]. About half of the intergenic nuclear enhancers transcribe eRNAs [17].

eRNAs are transcribed from an active enhancer by Pol II before transcription (Fig. 1). NamiRNA forms a complex with nuclear Argonaute 2 (nAGO2) and recruits p300, which catalyzes the acetylation of H3K27 (H3K27ac) at enhancers [18, 19] and activates other Pol II-recognized enhancer markers such as H3K4me3 and H3K4me1 [3]. Subsequently, Pol II recruits proteins such as TATA-box associated binding protein factor, positive transcription elongation factor b (P-TEFb), elongation factor PAF1 complex (PAF1), and SPT6 [20, 21] to bind to the active enhancer. Some of these TFs bind to other enhancer associated components such as p53 [11], P300 [22], CBP [23], mediator [24], YY1 [14], BRD4 [25], and cohesin [26]. The phosphorylation of Pol II C-terminal domain (CTD) and abundant poly (A) signals (PAS) immediately downstream of the transcription start site (TSS) create a correlation between the active enhancer transcription and Pol II stability [27]. This association leads to activation of Pol II, which first transcribes the enhancer region (with specific chromatin signatures such as H3K4me3, H3K4me1, and H2K27ac [28]), resulting in the production of eRNAs before transcribing other protein-coding genes (Fig. 1). The activated Pol II bidirectionally transcribes eRNAs at the active enhancer, resulting in both a sense and antisense eRNAs simultaneously [29] (Fig. 1). An integrator complex cleaves the 3ʹ UTR of the newly produced eRNA and trims it into appropriate sizes [9, 30].

The cleaving terminates transcription and unbinds eRNAs from Pol II, decreasing the transcript’s population at both strands [30]. eRNA transcription can also be halted by knocking out the enhancer’s promoter [17]. Following transcription termination, Pol II continues to transcribe neighboring genes in the enhancer loci. The RNA exosome degrades and regulates the transcribed eRNA population, resulting in its low abundance and reducing its ability to participate in pro-transcriptional processes and gene regulation [14, 31] (Fig. 1). eRNA biogenesis gives a clear understanding of the technical route of its biological function.

### NamiRNA is produced from miRNA coding genes with enhancer features

**Fig. 2:**
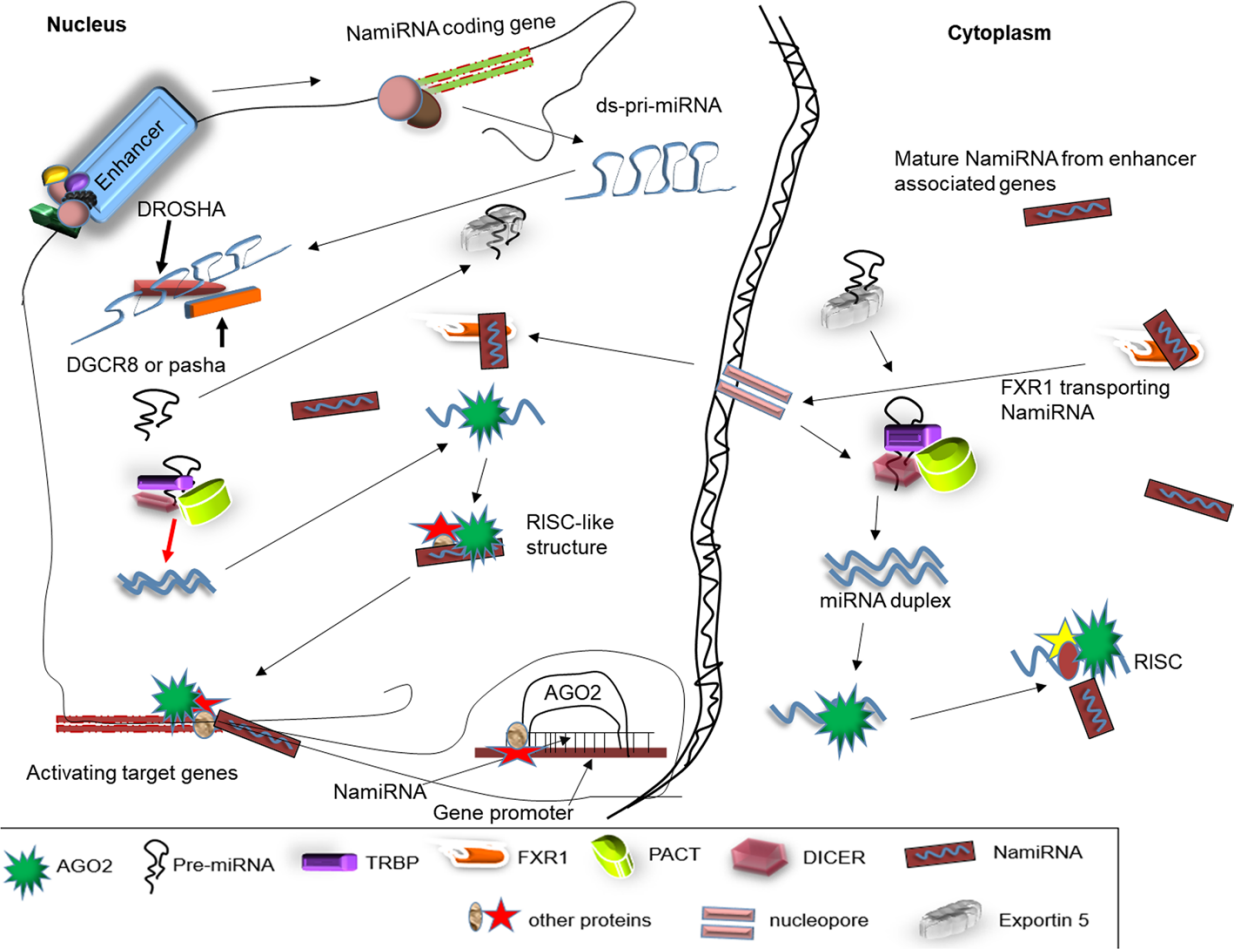
Biogenesis of NamiRNAs in the nucleus and cytoplasm. Pol II transcribes miRNA coding genes in the same loci as enhancers. MPC process the ds-miRNA into hairpin loop structures. Nuclear AGO2 and RNAi factors Dicer and TRBP process ds-miRNA into NamiRNAs in the nucleus. Alternatively, cytoplasmic produced miRNAs which were transcribed from genes with enhancer associated properties are activated by a RISC-like structure in the cytoplasm. The activated NamiRNAs in the cytoplasm are transported back into the nucleus via FXR1 to perform their activation and enhancing function

Nuclear AGO2 (nAGO2) and RNAi factors like Dicer and TRBP are proposed to process NamiRNAs in the nucleus with a similar mechanism as the canonical miRNA’s biogenesis [32]. During transcription, Pol II, after transcribing eRNAs, turns to transcribe miRNA-coding genes in enhancer loci. These genes and their miRNAs have enhancer features such as H3K4me1 and H2K27ac, which make them activators. Pol II transcribes these miRNA-coding genes into double-stranded primary miRNAs (ds-pri-miRNAs). With the help of specific ds-pri-miRNAs‑binding proteins, the microprocessor complex (MPC) (made up of Drosha and DGCR8) cleaves the ds-pri-miRNAs into smaller pre-miRNA hairpin loop structures named precursor RNAs [33, 34] (Fig. 2). The nDicer-nTRBP/PACT complex (nuclear Dicer binds to the TRBP/PACT) recognizes and cleaves the stem-loop off, forming mature double-stranded (ds) miRNA molecule in the nucleus [35]. The ds-miRNA then unwinds to release an nAGO2-bound single-strand RNA, which create a RISC-like structure with other proteins [37]. The structure guides the NamiRNA base pairing on enhancers or promoters of target genes to perform its activation (turning it into a NamiRNA) (Fig. 2).

Alternatively, NamiRNA may be produced in the canonical miRNA processing manner. Some pre-miRNA may escape the nuclear Ago2, Dicer, TRBP, and PACT and move to the cytoplasm with the transporter protein Exportin 5 through the nuclear pore. Upon reaching the cytoplasm, the pre-miRNA is cleaved into ds-miRNAs by the cytoplasmic Dicer-TRBP/PACT complex. The resulting ds-miRNA unwinds and releases a single strand to bind to AGO2. Together with AGO2, this strand binds to other proteins to form a RISC [36]. However, in the cytoplasm, matured miRNAs which were transcribed from genes with enhancer associated properties are activated by a similar RISC-like structure as in the nucleus. The activated NamiRNA then binds to Fragile X mental retardation syndrome-related protein 1 (FXR1), aiding it to shuttle between the nucleus and cytoplasm and subsequently activating the expression of nuclear genes and enhancers. These may explain how matured miRNAs in the cytoplasm can activate nuclear genes [37], and NamiRNAs can be produced in both the nucleus and the cytoplasm. The appearance of nucleus-located AGO2, Dicer, TRBP, and TRNC6A/GW182 confirms this hypothesis [32].

#### Uncertainties associated with eRNA biogenesis

Uncertainties seldom occur during eRNA transcription. However, diminution of the integrator subunits causes a decrease in eRNA’s signal-dependent induction and retracts stimulus-induced enhancer-promoter chromatin looping. These increase the spreading of GRO-seq reads throughout the body of eRNA transcripts at both enhancers and super-enhancers (SE) but make it difficult to detect [30]. Also, some H3K4me3 (an epigenetic marker of Pol II promoters) unmarked enhancers result in a transcriptional bias [7].

#### Uncertainties associated with NamiRNA biogenesis

There are some uncertainties related to the biogenesis of NamiRNAs. For example, Dicer-involved proteins, such as TRBP, can change the cleavage site of Dicer within some pre-miRNAs (e.g., pre-miR-132) [38]. Like pre-miR-451 in both zebrafish and mice [39], many miRNAs can escape cleaving by Dicer due to some extreme properties such as short sequences. Hence it is directly loaded into RISC for subsequent processing by AGO2 [39]. Recently, SE-mediated processing of associated pri-miRNA has been demonstrated. The deletion of several miR-1 or miR-290–295-related SE decreases their pri-mRNA production, possibly caused by reduced DGCR8 and Drosha recruitment, which affect miRNA processing and chromatin formation [40].

## eRNA and NamiRNA regulate transcription and gene activation

### eRNAs affect transcription during activation of their target

#### eRNAs activate target genes during chromatin looping

In the nucleus, eRNAs either activate or repress their target genes, and therefore their dysregulation affects transcription. For example, knockdown of specific eRNAs reduces its target genes’ expression, inferring their transcription roles [10]. Alternatively, exogenous eRNAs upsurge their prospective mRNA targets [41]. Nascent eRNA expression attenuates host gene expression during transcriptional elongation at intragenic enhancers [42]. Therefore, eRNA levels at a target gene correlate with its enhancers and neighboring genes [9]. Hence, they serve as additional drivers in inducing regulations in enhancer-regulated transcriptional controls [9].

eRNAs affect genes on the same or different chromosomes in a cis–trans mechanism (Fig. 4). This mechanism helps eRNAs to regulate target genes located either in front of or behind the enhancers. During its cis activity, eRNAs help activate their target in the same location as their topologically associated domain (TAD) with the eRNA loci [10, 17]. An example is ^DRR^eRNA; an eRNA transcribed from a distal enhancer of MyoD1 controls the expression of MyoD via a cis mechanism (Fig. 4). These eRNAs interact with the cohesion complex during trans activating myogenin (MyoG) expression [26] (Fig. 4). A distal eRNA knockdown is involved in chorionic gonadotropin alpha (Cga) genomic and chromosomal interactions [43].

Also, eRNAs can control their target genes’ expression by aiming at their promoter regions. Hence the loss of interaction between an eRNA and its promoter can cause a drastic change in orchestrating its target gene function and chromatin formation. Knocking down eRNAs triggers the replacement of histone H3K27ac merely at the enhancer by H3K27 trimethylation (H3K27me3) but not the promoter region [44]. Such situations lead to biased transcription of Pol II since it only recognizes promoters with H3K4me3, H3K4me1, and H2K27ac [28]. Also, the expression of eRNAs during the unwinding of DNA results in the formation of G quartets and R-loops, which causes genomic instability via the creation of three-stranded DNA–RNA hybrid loops [45]. These genetic recombinations and mutations are highly experienced in transcribed target genes compared to inactive transcriptional loci [46]. Conclusively, by assisting the target genes’ promoters’ activities and binding to sequence-specific DNA proteins, eRNAs control their targets' transcription mechanism, recapitulating their cell-specific gene expression and activation properties [8, 31]. Meanwhile, factors forming a complex with eRNAs can be identified by altering the transcript sequence or transcription itself [47].

#### eRNA controls genes with multiple eRNA-assigned regions and hnRNPL

Master TFs are examples of genes with multiple eRNA-assigned regions [48]. The distinct combinational module, including MyoD, MyoG, TCF12, TCF3, MEF2D, PBX1, and FoxO3, is involved in SE assembly and eRNA induction in myotubes [49]. These combinational modules with the multiple eRNA-assigned regions serve as a sponge for the eRNA. For example, loss of *MyoD* enhances the expression of typical enhancers-associated eRNAs (teRNA) and super-enhancer RNA (seRNA) in the myoblast [49]. These eRNAs then regulate the expression of other genes via an hnRNPL interaction. seRNA-1-hnRNPL interaction with the promoter and genic regions of neighboring genes, myoglobin (Mb) and apolipoprotein L6 increase Pol II, cyclin-dependent kinase 9 (CDK9), cyclin T1 (CCNT1), KMT3a, and H3K36me3 binding, inducing RNA expression [49]. Similarly, irregular expression of Bloodlinc and SERPINB2 eRNAs selectively upregulates their respective mRNAs [41].

### eRNAs associate with TF Yin-Yang in gene activation

Yin-Yang (YY)1 is a TF that regulates several genes’ transcription. It is recruited by regulatory element RNAs associated with promoter and enhancer sequences [14]. When halted, the abnormal RNA exosome causes an increase in eRNA accumulation but decreased YY1 recruitment to enhancers [14], impeding the transcription of the target genes of YY1 while increasing that of the eRNAs. During transcription, eRNA regulates transcription in YY1 activation in trans and cis [14].

## NamiRNA affects transcription while activating its targets

### NamiRNAs activate enhancers and eRNA transcription and alter chromatin looping

NamiRNAs have enhancer signatures (e.g., H3K27ac, P300/CBP, and DNase I high-sensitivity loci) and activate gene transcription as enhancer triggers [3, 19]. Over 400 miRNA precursors overlap histone modification markers, including H3K4me1 or H3K27ac on the UCSC browser [19], among which 303 miRNAs gene loci exhibit H3K27ac while the rest were within H3K4me1 [19]. A NamiRNA and its target enhancer activity become linked and function cooperatively when they associate; hence an enhancer’s activity is determined by its target NamiRNA expression pattern [40]. In the NamiRNA-enhancer regulation, enhancer-regulating NamiRNAs are mostly expressed around active enhancers. For example, miR-24-1 and miR-24-3p are around active enhancers and enriched H3K27ac regions [3]. Both NamiRNAs target their enhancers’ promoters and subsequently activate them, leading to the transcription of eRNAs [3].

Moreover, NamiRNA enhancer activation alters some of the enhancer properties, such as affecting transcription and chromatin looping when forming a complex with nAGO2 and Pol II and recruiting p300 to catalyze H3K27ac at activating enhancers [19]. For instance, miR-24 changes a chromatin state by increasing H3K27ac at its target enhancer [3]. Nevertheless, gene activation by NamiRNAs can only be achieved through an active and intact enhancer. This implies that altering an enhancer could result in an inactive or inhibited NamiRNA. Moreover, many NamiRNAs associating with enhancers are cell- and tissue-specific [50]. For example, H3K27ac SEs mark many tissue-specific master miRNAs [40]. Therefore, using its enhancer associated markers or properties such as H3K27ac would be an easy and efficient method to locate NamiRNAs during subsequent research.

### NamiRNA activates target genes during transcription

NamiRNAs mainly orchestrate target enhancers and gene activation [3]. The human miRNA 373 was the first nuclear RNA discovered to possess gene activation properties by affecting the transcription of E-cadherin (CDH1) and cold-shock domain-containing protein 2(CSDC2) [51]. NamiRNA has a similar sequence as eRNAs; hence they perform the same activities (e.g., gene activation) with similar mechanisms in cis or trans [9]. The NamiRNA miR-26a-1, when overexpressed, activates the transcription of its neighboring genes integrin alpha-9 (ITGA9) and Villin-like (VILL) located in a 400 kb window [3]. Similarly, the NamiRNA miR-339 upregulates its neighboring gene, G protein-coupled estrogen receptor 1 (GPER), by fourfold [3]. NamiRNA sometimes controls the target gene by inhibiting or activating its upstream suppressor and promoters sharing its transcriptional factors. NamiRNA miR-205 activates tumor suppressor genes interleukin 24 (IL24) and interleukin 32 (IL32) via targeting specific sites in their promoters [52]. In NamiRNA transcriptional gene activation (TGA), nuclear proteins such as nAGO2, nDicer1, and Gw182/TNRC6 assist NamiRNAs in the modification of chromatin, enrichment, and regulation of Pol II and gene promoters [18, 53]. An example of other mechanisms involved in NamiRNAs’ TGA is an interaction with ribosomal proteins in the 5ʹ UTR of the mRNA to induce gene translation by miR-10a [54]. Conclusively, NamiRNAs serve as gene and enhancer transcription triggers [3] and regulators [55].

### Model of NamiRNA activation

During TGA, NamiRNAs recruit an nRISC-like structure (containing up to 7 nt) [56] and bind to a complementary sequence on a promoter via their seed regions. The complex cleaves the antisense non-coding transcript and releases the transcriptional repression complex, making the target genes free and activated for transcription [57]. Alternatively, the NamiRNA-nAGO-protein complex (consisting of transcriptional activators) binds to the gene promoter region or the 5ʹ UTR of a nascent RNA (or a pRNA), modifying the chromatin structure into a more proper shape to induce gene transcription [58]. In the nucleus, the targets of NamiRNAs include ncRNA, pri-miRNA, promoter, and enhancer [59].

#### NamiRNAs can regulate their population in the nucleus and associate with ribosomal RNA in the cytoplasm

NamiRNAs are stored and exist as pri-miRNA and mature miRNAs in the nucleus [3] and can regulate the miRNA population. An example is the mature let-7 NamiRNA [60] in *Caenorhabditis elegans*, which binds to its pri-miRNA to form a positive feedback loop during its processing [61]. The mouse miR-709 binds to the 19-nt recognition element on pri-miR-15a/16-1, preventing them from processing [62]. This contributes to the concept that the maturity of some miRNAs/NamiRNAs depends on splicing factors [63] and positive feedback looping of other miRNAs/NamiRNAs. Hence controlling a miRNA’s production using its mature miRNA can be productive. NamiRNAs also regulate the abundance of rRNAs by interacting with ribosomal subunits (28S and 45s rRNA). miR-206 interacts with 28S ribosomal RNA (rRNA) in the nucleolus and the cytoplasm of mammalian cells [64], which affects the abundance of rRNA and helps the ribosomes express properties needed for interactions with their binding proteins [65]. The miRNA-nRISC is believed to work as a defense mechanism [66], but it has not yet been fully exploited. The cellular activity similarities between eRNA and NamiRNA in gene activation are summarized in Table 1.

**Table 1:** Cellular activity similarities between eRNA and NamiRNA

Activities	eRNA		miRNA	
	Cytoplasm	Nucleus	Cytoplasm	Nucleus
Cellular stage of operation	No evidence yet	Pre-transcription	Post-transcription	Post-transcription, transcriptional
Location	No evidence yet	Yes	Yes	Yes
Target	No evidence yet	Promoter sequence [44], enhancers [9], DNA [45], lncRNA, and other ncRNA [67]	mRNA [68]	Pri-mRNA [61], promoter [59], enhancer [3], ncRNA [59]
Transcriptional effect	No evidence yet	Activation [48], silencing [10]	Activation [54]	Activation [3]
Mode of action	No evidence yet	RNA–RNA hybrid RNA–DNA-DNA hybrid [45]	RNA-RNA hybrid [69]	RNA–RNA hybrid RNA–DNA hybrid RNA–DNA–DNA hybrid [69]
Cis and trans activities	No evidence yet	Yes [67]	No evidence yet	Yes [70]
Transcriptional activity	No evidence yet	Yes	Yes	Yes
Activation of genes/mRNA	No evidence yet	Yes [48]	No	Yes

## eRNA and NamiRNA co-function in muscle development

### eRNA and NamiRNA regulate myogenic regulatory factors, TFs, and myogenic genes during myogenesis

**Fig. 3:**
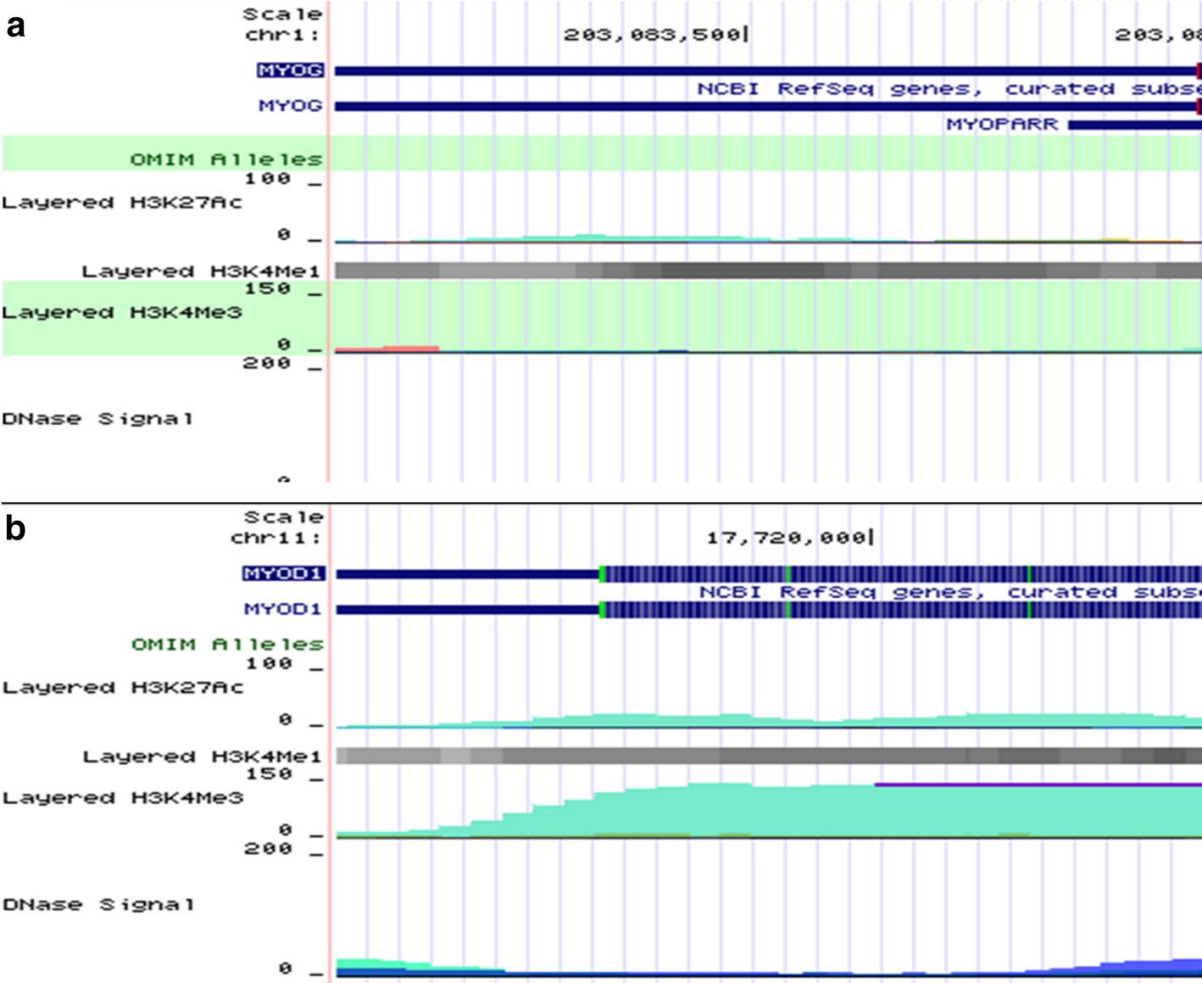
Myogenic genes MyoG (**a**) and MyoD (**b**) exhibit enhancer features during myogenesis. MyoD (chromosome chr11:17,719,571–17,722,136; 2,566 bp) and MyoG (chr1:203,083,129–203,086,012; 2,884 bp) exhibits active enhancer signatures such as high H3K4me1-to-H3K4me3 ratio and H3K27ac [[85]. Source: The UCSC Genome Browser database: 2015 update, Dec. 2017 patch release 12. http:// genome-asia.ucsc.edu . MYOD1 (ENST00000250003.4) at chr11:17,719,571–17,722,136—Homo sapiens myogenic differentiation 1 (MYOD1), mRNA. (Ref Seq NM_002478), MYOG (ENST00000241651.5) at chr1:203,083,129–203,086,012—Homo sapiens myogenin (MYOG), mRNA. (from Ref Seq NM_002479); DEC.2013 (GRCh38/hg38)]

**Fig. 4:**
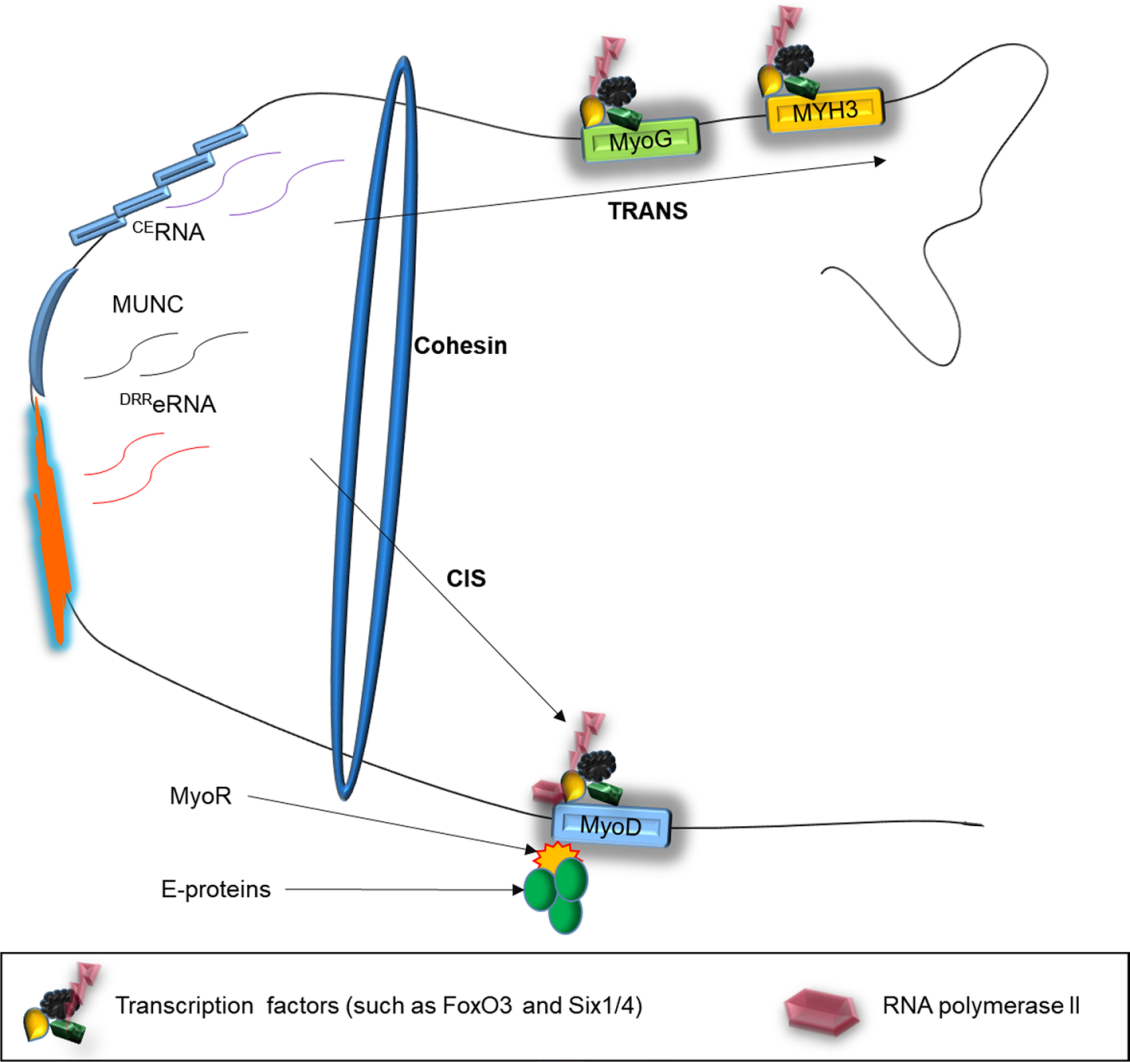
CERNA, MUNC, and DRReRNA associate with MyoD and MyoG in cis and trans during myogenesis. MyoD expression is induced by the TFs FoxO3, Six1/4, and Pax3 and Pax7 and regulated by CERNA in cis. However, DRReRNA in trans via interacting with cohesin complex activates MyoG. MUNC via a heterologous promoter in trans promotes endogenous gene expression of MyoD, myogenin, and Myh3. bHLH protein MyoR binds E-proteins directly on MyoD target DNA sequences hence repressing its activities

^CE^RNA and ^DRR^eRNA enhance skeletal muscle cell differentiation and Pol II residency at MyoD and MyoG loci, respectively [73] (Fig. 4). ^DRR^eRNA exhibits enhancer function in early differentiation while ^CE^RNA is transcribed in proliferating myoblasts [48]. During myogenesis ^DRR^eRNA in trans via interaction with cohesin complex activates MyoG expression and the rest of the myogenic gene regulatory network without influencing MyoD transcript levels [48]. ^DRR^eRNA recruits nascent transcripts of MyoG and associates with protein complexes involved in eRNA biogenesis, such as Integrator and WD Repeat Domain 82 (WDR82) [30]. Following that, ^DRR^eRNA via the formation of RNA:RNA interaction or via an RNA/DNA triple helix at the MyoG locus recognizes target intronic regions of the MyoG nascent transcripts [26]. After safely binding to MyoG via these mechanisms, ^DRR^eRNA then represses the expression of MyoG in trans. Simultaneously, ^DRR^eRNA safeguards proper cohesin loading in trans to regulate gene expression and increase chromatin accessibility. A transcribed eRNA from an enhancer region on mouse chromosome 7 (eRNA) recruited cohesin to regulate the MyoG gene’s transcription on chromosome 1 [26].

Myogenesis is controlled by a network of epigenetic regulators and transcriptional factors. The myogenic regulatory factor (MRF) members, including myogenic factor 5 (Myf5), MyoD, MyoG, and myogenic regulatory factors (MRF4), are responsible for orchestrating myogenesis. Other myogenic genes such as Paired box gene 3 (Pax3) and Paired box gene 7 (Pax7) mark the presence of these muscle progenitors [71]. Though not all satellite cells express Pax3 and Pax7 in the postnatal myofiber, they activate proliferation-related genes inhibiting differentiation [71]. MyoD and MyoG have similar genome-wide binding profiles in myogenesis that may display enhancer or eRNA features [48]. MyoD controls many myogenic eRNAs, while silencing MyoG affects their expression mildly [48]. MyoD expression in proliferating myoblast is induced by TFs, including FoxO3, Six1/4, Pax3, and Pax7 [72] (Fig. 4). However, MyoD and MyoG exhibit active enhancer signatures (high H3K4me1/H3K4me3 ratio, acetylated histones, and polymerase II-occupied) bound by Pol II and generate RNA (Fig. 3a, b). These aid MyoD in regulating its core enhancer RNA (^CE^RNA) and associated enhancer expression. Alternatively, ^CE^RNA activates MyoD expression in cis and ^DRR^eRNA (also named MUNC) in trans induces MyoG transcription and muscle differentiation (Fig. 4), suggesting transverse regulation between them [67].

Moreover, NamiRNAs enhance some myogenic genes in their myogenic activities. For example, the conserved miR-675-3p and miR-675-5p encoded by exon 1 of H19 empowers H19 to enhance myogenic differentiation [74]. The former performs the above function by suppressing BMP pathway TFs, Smad1, and Smad5. The latter suppresses levels of cell division cycle 6 (Cdc6), a DNA replication initiation factor [74]. The knockdown of activin receptor type-2B (ACVR2B) using 5ʹ UTR and 3ʹ UTR derived muscle creatine kinase (MCK) promoter-driven artificial microRNAs (amiRNAs) caused a decrease in MAD2/3 signaling and SMAD2/3 signaling, respectively. The latter reduces MRFs’ expression and suppresses myogenesis while the former induced decreased MAD2/3 signaling, increased MRF expression, and enhanced proliferation and differentiation of myoblasts in goats [75]. A vivid mechanism of miRNAs’ regulatory mechanisms in muscle development and diseases has been reviewed [76].

### Some lincRNAs mimic eRNA

Apart from ^CE^RNA and ^DRR^eRNA, some lncRNAs regulate myogenesis [77–80] via orchestrating their neighboring genes independent of their sequence [67]. For example, LncMyoD enhances myogenesis and represses the translation of proliferation genes (N-RAS and c-Myc) via IMP2 (an IGF-2 mRNA binding protein) while functioning as eRNAs [81]. The muscle-specific lncRNA (linc-MD1) associate with Duchenne muscular dystrophy (DMD) to control the expression of transcriptional factors mastermind-like protein 1 (MAML1) and MEF2C [82]. LincRNA Yam-1 represses Wnt7b and activates miR-715 to regulate myogenic differentiation [83].

## NamiRNAs colocalize with enhancers and eRNAs

### Proposed eRNA and NamiRNA dual gene activation model

**Fig. 5:**
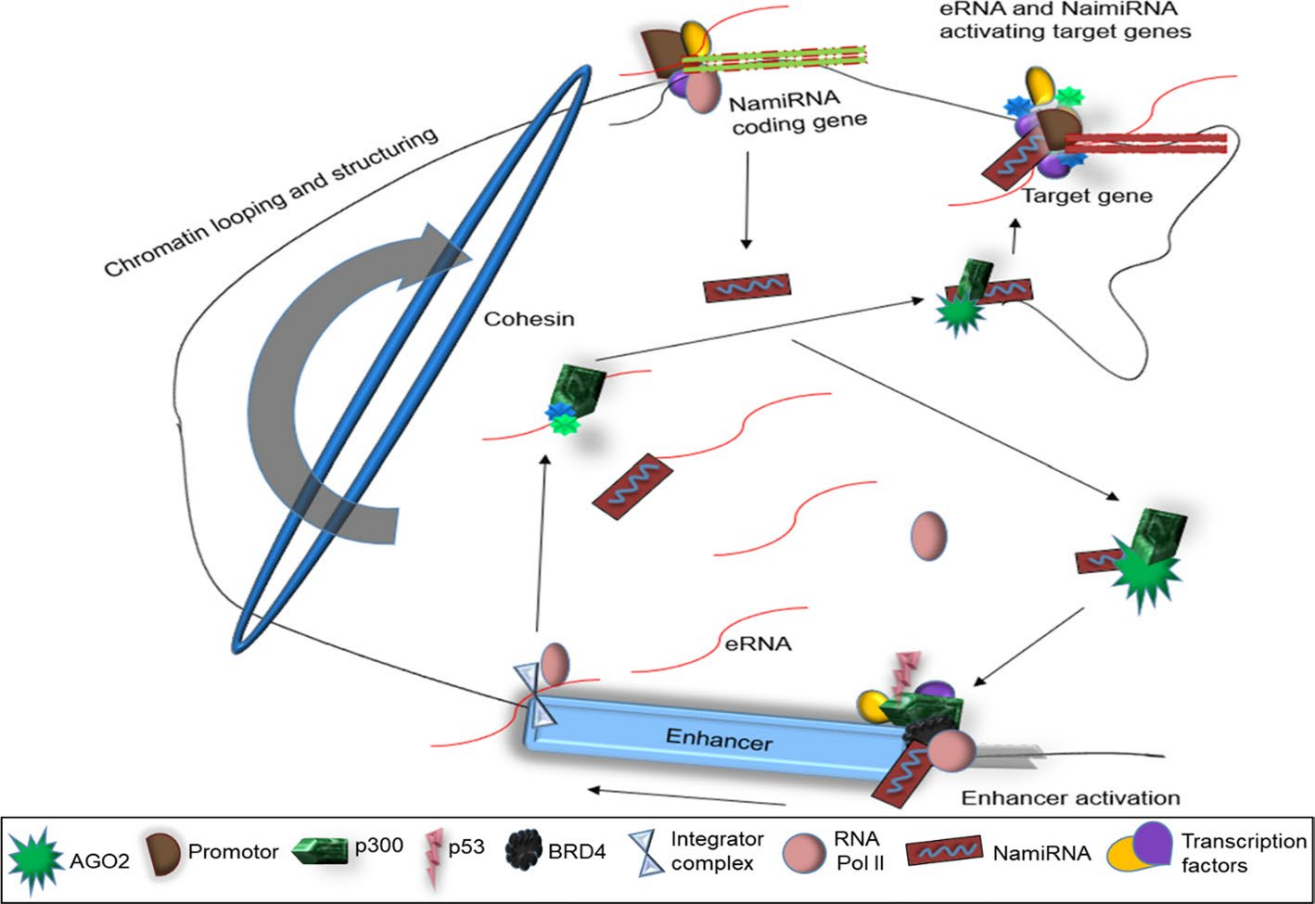
The proposed model of eRNAs and NamiRNAs cooperatively enhancing and activating target genes. NamiRNAs form a complex with AGO2 and p300; they bind to enhancers and activate markers such as H3K27ac and H3K27me3, which attract RNA polymerase to recognize and translate the enhancer. Transcribed RNAs bind to p300 and other proteins to activate enhancer features at the target gene’s promoters. The attracted NamiRNAs then attach to the gene’s promoter and subsequently activate the target gene’s expression

eRNAs and NamiRNAs work simultaneously to activate target genes in this proposed model. Chromatin looping and high alteration of chromatin structure before replication proximate target genes and enhancers of both NamiRNA and eRNA, inducing an interaction between the enhancers and genes of eRNAs and NamiRNA. NamiRNAs target the enhancer promoters and subsequently activate them, leading to the transcription of eRNAs [3]. NamiRNA forms a complex with nAGO2 and Pol II and activates markers such as H3K27ac, H3K4me3, and H3K4me1 at active enhancers [18, 19]. The enhancer’s activation is recognized by Pol II, which then transcribes eRNAs [3] (Fig. 5). The eRNAs interacting with CBP and p300 [23] elevate chromatin accessibility for TFs via acetylating histones H3 and H4 at the gene promoter [84]. These activities of eRNAs alter enhancer features such as H3K27ac and DNase hypersensitivity and the binding of TFs [85]. For example, knockdown of eRNAs at their respective enhancer and target-promoter areas decreases H3K27ac but increases H3K27me3 levels [43]. These enhance features at genes’ promoter and attract NamiRNAs. The attracted NamiRNAs overlap within the enhancer markers and form a complex with nAGO2 and recruits p300, catalyzing H3K27ac at the promoter region and activating it (Fig. 5). These predict an interactive eRNA-NamiRNA, starting and improving target genes on the same or different chromosomes during chromatin looping [10, 17]. Transcribed eRNAs may orchestrate miRNA expression since eRNAs can solely regulate gene expression [73].

### eRNAs and NamiRNAs located on different chromosomes may interact during their activities

**Fig. 6:**
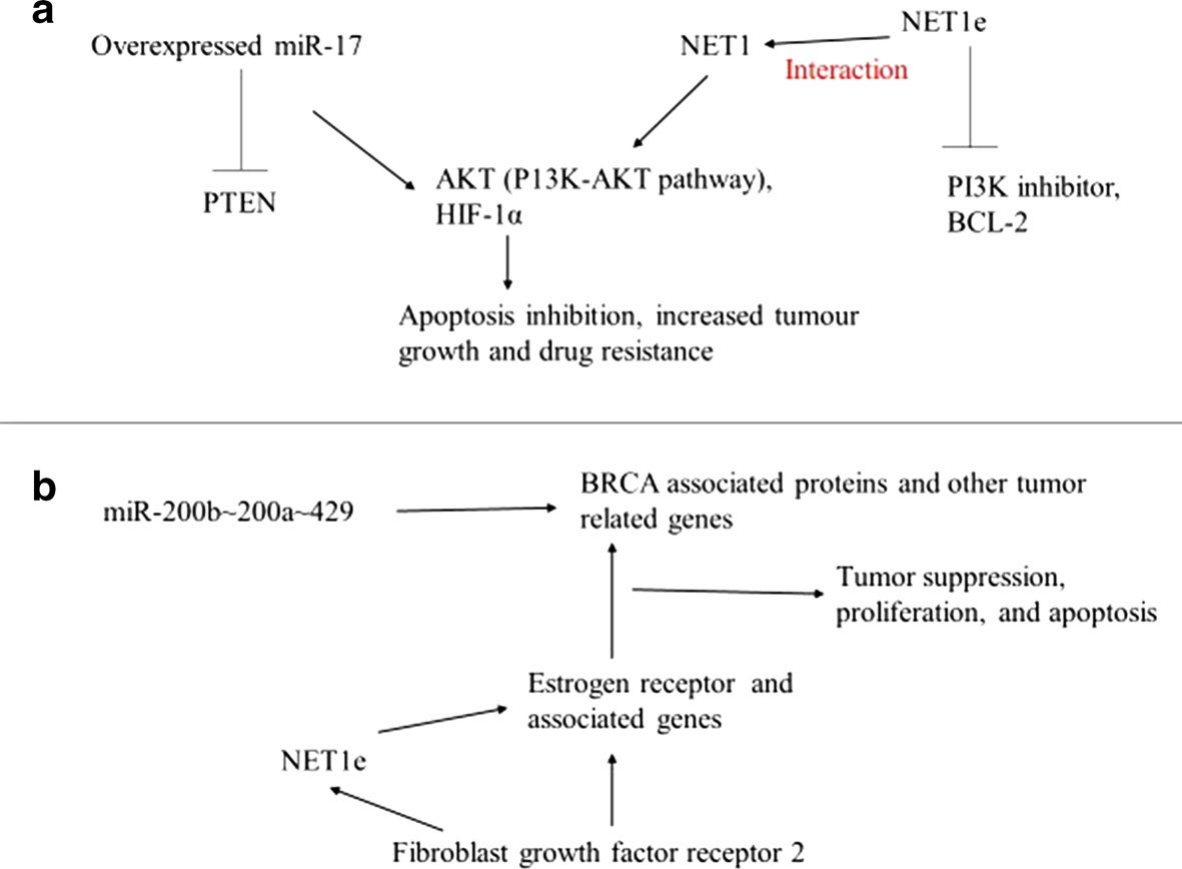
Proposed eRNA and NamiRNA interaction in drug resistance (**a**) and breast cancer tumorigenesis (**b**). During drug resistance, overexpressed miR-17 represses PTEN leading to the activation of the PI3K-Akt pathway and HIF-1α. Simultaneously, Net1e increases the expression of NET1 while inhibiting the PI3K inhibitor and BCL-2. NET1 and NET1e interact with miR-17 via the PI3K-Akt pathway to inhibit apoptosis and increase tumor growth, while NET1 causes induced drug resistance (**a**). In breast cancer, fibroblast growth factor receptor 2 interacts with NET1e, the Estrogen receptor (ER), to regulate BRCA and other cancer genes, which are targets of miR-200b ~ 200a ~ 429. These trigger an interaction between miR-200b ~ 200a ~ 429, ER, NET1e, and ER-associated genes leading to tumor suppression, increased proliferation, and inhibited apoptosis in breast cancer cells

NamiRNAs and eRNAs located on different chromosomes may interact and perform similar functions. For example, miR-17 from miR-17HG interacts with NET1e, an eRNA transcribed from a close enhancer of the NET1 gene to induce drug resistance (Table 2). Overexpressed miR-17 knocks down PTEN (its target tumor suppressor), activating other downstream cellular components such as AKT and hypoxia-inducible factor-1α (HIF-1α) [86]. Similarly, NET1e interacts with the NET1 to form subsequent resistance to induced compounds and drugs, hence worsening cell survival and increasing tumor growth [87]. And overexpressing NET1e caused drug resistance to the PI3K inhibitor and BCL-2 inhibitor in MCF7 cells via PI3k-Akt pathways [87]. It can be speculated that NET1e can function similarly to miR-17 since both exhibit Akt pathways during their drug resistance activities, indicating an interaction between them (Fig. 6a).

**Table 2:** Some diseases and therapeutics involving both miRNA and eRNA

Diseases and therapeutics	miRNA	eRNA
General cell signaling pathway	miRNAs [75]	NET1e [87]
MAP kinases and NFkB	miR-22 and miR-140 [91]	ADAMDEC1 eRNA[22]
Adenocarcinoma	miR-3131, miR-664, miR-483 and miR-150 [92]	NET1e, general eRNAs [87]
Cancer	miR-375[93], miR-137 [94]	eRNAs of BRCA [87]
Prostate cancer	miR-373 [51]	KLK3e [10]
Tumor suppression	miR-17-92 [89]	p53BERs eRNAs [11]
Apoptosis and tumorigenesis	miR-129-2 [95], miR-495 [96], microRNA-378 [97]	lnc-SLC4A1-1 [98] general eRNAs[99]
Immunity	miR-212 [100]	AP001056.1 [101]
Breast cancer	miR-200b ~ 200a ~ 429 [90]	miR-200b eRNA [90], NET1e [87] estrogen receptor α (ER-α)-bound eRNA( +) [102]
Drug resistance	miR-17 [86]	NET1e [87]

Moreover, NamiRNAs and eRNAs contribute to cancer drug resistance through enhanced drug efflux, altered drug metabolism, and enhanced anti-apoptosis pathways [88]. In tumor suppression and apoptosis, eRNAs originating from p53-bound enhancer regions (p53BERs) are required for p53-dependent cell-cycle arrest; NamiRNA miR-17–92 suppresses chromatin regulatory genes (Sin3b, Hbp1, Suv420h1, and Btg1) and the apoptosis regulator (Bim) via similar TFs (such as AGO2 and FOXA1) to regulate cell survival and autonomous proliferation [89]. Another example of eRNA-NamiRNA dual interaction is the proposed model of Net1e and miR-200b ~ 200a ~ 429 in breast cancer. The miR-200b ~ 200a ~ 429 gene produces miR-200b eRNAs from an enhancer located approximately 5.1 kb upstream [90]. miR-200b ~ 200a ~ 429 targets BRCA associated proteins and orchestrates their expression in cancer. Moreover, fibroblast growth factor receptor 2 (FGFR2) interacts with NET1e and the estrogen receptor (ER); ER associates with BRCA and other cancer genes that are targets of miR-200b ~ 200a ~ 429. These connect miR-200b ~ 200a ~ 429, miR-200b eRNA, NET1e, and ER-associated genes, leading to breast cancer regulation by controlling tumor suppression, proliferation, and apoptosis (Fig. 6b). The activities and expression of these RNAs can be a predictive factor in monitoring the expression of genes associated with the eRNAs mentioned above in breast cancer. These observations demonstrate that chromatin looping aids the interaction between eRNAs and NamiRNAs, which associates and works hand in hand to regulate cellular activities.

#### eRNA and NamiRNA action in disease diagnosis

Recently, SEs, enhancers, and eRNAs have been used as factors for analyzing, mapping, and studying a broad range of conditions, including autoimmunity [103], cancer [101], and muscle-related disease such as muscular dystrophies [104]. About 80% of genes in the canonical cancer signaling pathways are associated with specific eRNAs [87] and NamiRNAs in at least one cancer type. A genome-wide association study found eRNAs or SEs near known genetic variants for autoimmune disease risk in autoimmune disease patients, hence serving as biomarkers [103], which makes disease diagnostics easy [22, 109].

Moreover, eRNAs and NamiRNAs explore autophagy, apoptosis, and signaling pathways to regulate diseases. For instance, overexpression of the NamiRNA miR-378/378 enhances autophagy and represses apoptosis by targeting caspase 9, while the opposite reduces autophagy and accumulates abnormal mitochondria, and enhances apoptosis. These miRNAs target the rapamycin (mTOR)/unc-51-like autophagy activating kinase 1 pathway to inhibit apoptosis, and target phosphoinositide-dependent protein kinase 1 to maintain autophagy via Forkhead box class O (FoxO)-mediated transcriptional reinforcement [97]. Alternatively, the knockdown of growth-regulating estrogen receptor binding 1 (GREB1) eRNA enhances apoptosis and represses proliferation in bladder cancer [105]. These small RNA molecules, i.e., NamiRNA and eRNA, are predicted to contribute to cancer drug resistance through enhanced drug efflux, altered drug metabolism, overexpression of target molecules, and enhanced survival anti-apoptosis pathways [88]. Subsequent downregulation of NamiRNAs miR-34, miR-17, and let-7a is associated with sensitivity to drugs commonly used as cancer treatments.

### eRNA and NamiRNA activities in therapeutics

NamiRNA and eRNA are the next options in diagnosing, treating, and studying the pathogenesis of diseases that are not associated with current biomarkers [106]. People can exploit these biomarkers regarding the dysregulation of genes and mRNAs in myopathy and other disorders. Concerning this effort, the USA FDA approved its first siRNA drug (patisiran infusion) in 2018 to treat peripheral nerve disease caused by hereditary transthyretin-mediated amyloidosis [107]. Moreover, the drug’s therapeutic mechanism is based on silencing the RNA that causes the disease. eRNAs are now known to play roles in diseases such as myopathy; hence eRNA-targeted therapy [108] may be the next option. However, the environmental conditions or manipulation affects the expression and activities of NamiRNAs and eRNAs in diseases. Likewise, epigenetic factors also regulate eRNAs and NamiRNAs. For instance, epigenetic silencing of cell or tissue-specific eRNAs or NamiRNAs (e.g., miR-495 [96], Epstein–Barr virus super-enhancer eRNAs [109], and miR-335 [110]) can induce proliferation. Table 2 presents some diseases and therapeutics involving both eRNA and NamiRNA.

## Conclusion

Briefly, NamiRNAs activate enhancers to initiate eRNA transcription. The transcribed eRNAs then interact with NamiRNA through chromatin looping to orchestrate the expression of their target genes via similar TFs and mechanisms such as eRNA-NamiRNA dual gene activation, enhancer-promoter interactions, chromatin looping, and signaling pathways. eRNAs and NamiRNA use a similar tool in their activities to associate with enhancers and their target genes, which gives them functional similarities concerning their association with myogenesis (Table 1), myopathy, and therapeutics (Table 2). NamiRNAs and eRNAs such as MUNC, ^CE^RNA, and ^DRR^eRNA effectively regulate myogenic genes and factors during myogenesis (Fig. 4). Hence, we deem eRNAs as functional molecules, transcriptional regulators, and partners of NamiRNAs.

The building of data portals for biomolecular markers such as eRic for eRNAs [87] can be used to provide prognostic markers for the future prediction of disease risk and progression. Moreover, the adverse effects of eRNA and NamiRNA inhibitors and other RNAs can enhance myogenesis, diseases, and therapeutics; hence exploring their genetic alterations will benefit both humans and animals. Using CRISPR /Cas 9, TALENS, and other modern genetic editing tools, several options help to unveil NamiRNA- and eRNA-associated regulations. These can be applied in therapeutics related to myogenesis and transcription. However, safety issues, including off-target effects and confirmation of the proposed models in this review, still need to be addressed.

Some current challenges associated with eRNA and NamiRNAs need to be explored to understand these molecules better. Firstly, most proposed models have not been tested; hence the field requires more research. Some enhancers lack H2K27ac; this results in Pol II biased transcription; therefore, a solution to this problem would be beneficial [28]. Moreover, cellular pathways of diseases expressing the proposed interaction of NamiRNA and eRNAs need to be studied to confirm these models; perhaps there is more to it than we know. Also, monitoring the interaction between eRNA and NamiRNAs is difficult; hence an in vivo imaging system or other bioluminescence methods need to monitor their interaction during chromatin looping and gene activation. However, there is no database designed to document and map eRNA-NamiRNA interactions with genes and enhancers. There are still some misconceptions about the proteins responsible for NamiRNA processing; thus identifying all the cellular molecules that may regulate the processing and activation of NamiRNAs in the nucleus and the transportation of NamiRNAs from the cytoplasm to the nucleus is necessary. Lastly, it is essential to determine whether the NamiRNA uses the same seed sequence to activate target enhancers and genes.

## Data Availability

The datasets generated and/or analyzed during the current study are available in The UCSC Genome Browser database: 2015 update, Dec. 2017 patch release 12. http:// genome-asia.ucsc.edu [111] repository [source for Fig. 3 A (MYOD1 (ENST00000250003.4) at chr11:17,719,571–17,722,136 – Homo sapiens myogenic differentiation 1 (MYOD1), mRNA. (Ref Seq NM_002478), B (MYOG (ENST00000241651.5) at chr1:203,083,129–203,086,012 – Homo sapiens myogenin (MYOG), mRNA. (from Ref Seq NM_002479)); DEC.2013 (GRCh38/hg38)].

## Abbreviations

ACVR2B: Activin receptor type-2B
ADAMDEC1: ADAM-like Decysin 1
AGO2: Argonaute 2
amiRNAs: Artificial microRNAs
Apol6: Apolipoprotein L6
BAPB: BRCA1 associated protein
BCCIP: BRCA2 and CDKN1A interacting protein
BDs: Broad domains
bHLH: Basic helix-loop-helix
BRD4: Bromodomain-containing protein 4
BRDs: Extra-terminal motif (BET) proteins
BRIP1: BRCA1 interacting protein C-terminal helicase 1
CCNT1: Cyclin T1
Cdc6: Cell division cycle 6
CDH1: E-cadherin
CDK9: Cyclin-dependent kinase 9
CERNA: Core enhancer RNA
Cga: Chorionic gonadotropin alpha
CRISPR: Clustered regularly interspaced short palindromic repeats
CSDC2: Cold-shock domain-containing protein 2
CTD: C-terminal domain
CTRP: Cancer Therapeutics Response Portal
CTX: Cardiotoxin
DMD: Duchenne muscular dystrophy
DRReRNA: Distal regulatory regions RNA
ds-pri-miRNA: Double-stranded primary miRNA
E:P: Enhancer:promoter
ER: Estrogen receptor
eRNA: Enhancer RNA
ER-α: Estrogen receptor α
FoxO: Forkhead box class O
FOXO1: Forkhead box protein O1
FoxO3: Forkhead box protein O3
FXR1: Fragile X mental retardation syndrome-related protein 1
GPER: G protein-coupled estrogen receptor 1
GREB1: Growth-regulating estrogen receptor binding 1
hnRNPL: Heterogeneous nuclear ribonucleoprotein L
HuR: Human antigen R
IL24: Interleukin 24
IL32: Interleukin 32
ITGA9: Integrin alpha-9
linc-MD1: Muscle-specific lncRNA
lncRNA: Long non-coding RNA
m½-sbsRNAs: Mouse Staufen1-binding site RNAs
Malat1: Metastasis-associated lung adenocarcinoma transcript 1
MAML1: Mastermind-like protein 1
MAP kinase: Mitogen-activated protein kinase
Mb: Myoglobin
MCK: Muscle creatine kinase
MEF2A: Myocyte-specific enhancer factor 2A
MEF2C: Myocyte-specific enhancer factor 2C
MEF2D: Myocyte-specific enhancer factor 2D
miRNA: Micro RNA
MRF4: Myogenic regulatory factor 4
MRFs: Myogenic regulatory factors
mTOR: Mammalian target of rapamycin
Myf5: Myogenic factor 5
MyoD1: Myoblast determination protein 1
MyoG: Myogenin
nAGO2: Nuclear Argonaute 2
NamiRNA: Nuclear activating miRNA
ncRNA: Non-coding RNA
nDicer: Nuclear Dicer
NELF: Negative elongation factor
NET1e: NET1 enhancer RNA
nRISC: Nuclear RISC
nt: Nucleotides
nTRBP: Nuclear TRBP
nTRBP/PACT: Nuclear TRBP/PACT
p53BERs: P53 bound enhancer RNAs
PAF: PAF1 complex
PAS: Poly(A) signals
Pax3: Paired box gene 3
Pax7: Paired box gene 7
PBX1: Pre-B-cell leukemia TF 1
PDK1: Phosphoinositide-dependent protein kinase 1
Pitx2: Paired-like homeodomain factor 2
PRC2: Polycomb repressive complex 2
pRNA: A nascent RNA or a non-coding promoter transcript
P-TEFB: Positive transcription elongation factor b
RISC: RNA-induced silencing complex
RNA Pol II: Pol II
RNase III: RNA ribonuclease III
rRNA: Ribosomal RNA
SE: Super-enhancers
seRNA: Super enhancer RNA
SERPINB2: Serpin family B member 2
snRNAs: Nuclear RNAs
SPT6: Transcription elongation factor SPT6
SRA: Steroid receptor RNA activator
TAD: Topologically associating domain
TALEN: Transcription activator-like effector nuclease
TCF12: TF 12
TCF3: TF 3
teRNA: Typical enhancers associated eRNAs
TF: Transcription factors
TGA: Transcriptional gene activation
TGS: Transcriptional gene silencing
TRBP: Transactivation-response RNA-binding protein
TSS: Transcription start site
ULK1: Unc-51-like autophagy activating kinase 1
UTR: Untranslated region
VILL: Villin like
WDR82: WD repeat domain 82
YYI: Yin-Yang I
